# Increased neuronal death and disturbed axonal growth in the Polμ-deficient mouse embryonic retina

**DOI:** 10.1038/srep25928

**Published:** 2016-05-13

**Authors:** Jimena Baleriola, Noemí Álvarez-Lindo, Pedro de la Villa, Antonio Bernad, Luis Blanco, Teresa Suárez, Enrique J. de la Rosa

**Affiliations:** 1 3D Lab (Development, Differentiation and Degeneration), Centro de Investigaciones Biológicas, CSIC, 28040 Madrid, Spain; 2 System Biology Department, School of Medicine, Universidad de Alcalá, 28802 Alcalá de Henares, Spain; 3 Centro Nacional de Biotecnología, CSIC, 28049 Madrid, Spain; 4 Centro de Biología Molecular Severo Ochoa, CSIC-UAM, 28049 Madrid, Spain

## Abstract

Programmed cell death occurs naturally at different stages of neural development, including neurogenesis. The functional role of this early phase of neural cell death, which affects recently differentiated neurons among other cell types, remains undefined. Some mouse models defective in DNA double-strand break (DSB) repair present massive cell death during neural development, occasionally provoking embryonic lethality, while other organs and tissues remain unaffected. This suggests that DSBs occur frequently and selectively in the developing nervous system. We analyzed the embryonic retina of a mouse model deficient in the error-prone DNA polymerase μ (Polμ), a key component of the non-homologous end-joining (NHEJ) repair system. DNA DSBs were increased in the mutant mouse at embryonic day 13.5 (E13.5), as well as the incidence of cell death that affected young neurons, including retinal ganglion cells (RGCs). Polμ^−/−^ mice also showed disturbed RGC axonal growth and navigation, and altered distribution of the axonal guidance molecules L1-CAM and Bravo (also known as Nr-CAM). These findings demonstrate that Polμ is necessary for proper retinal development, and support that the generation of DSBs and their repair via the NHEJ pathway are genuine processes involved in neural development.

During neural development diverse cellular processes, including proliferation, neurogenesis, migration, axonogenesis and synaptogenesis, need to be exquisitely coordinated in order to achieve proper nervous system structure and function. Programmed cell death, besides refining neuronal number and connectivity, also occurs at early developmental stages, exerting a greater impact on neural physiology than initially thought^1^,^2^,^3^,^4^,^5^.

Cues on the origin of that early phase of neural cell death arise from the study of mouse models defective in DNA repair. DNA double-strand breaks (DSBs) activate two complementary DNA repair pathways: homologous recombination (HR), which favours genomic stability and fidelity, and non-homologous end joining (NHEJ), which is error-prone^6^,^7^,^8^,^9^. Remarkably, NHEJ is the only DSB repair pathway active in postmitotic cells, as neurons are. Mouse models defective in HR or NHEJ show massive cell death in the developing nervous system, even leading to embryonic lethality, while other tissues are minimally affected^6^,^10^,^11^,^12^. These observations reveal that the central nervous system is particularly sensitive to DSBs during its development^13^. Further, DNA repair is essential to ensure genomic integrity and to preserve neural cell homeostasis^14^,^15^.

The origin of the DSBs underlying the selective neural phenotype in models of defective DSB repair remains unclear. DNA damage may be a consequence of replication stress during proliferation, transcriptional activity, or oxidative stress produced by normal metabolism^15^. The high proliferative rate during nervous system development, as well as the postmitotic condition of neurons, may explain the sensitivity of the nervous system to DSBs during development and aging, as well as in pathological conditions^13^,^15^. However, this fact alone cannot easily explain the selective neural, as well as immunological, phenotype displayed by mouse models of defective DSB repair, while other highly proliferative and metabolically active tissues are less affected^2^,^11^,^12^,^16^.

To further analyze the relationship among neurogenesis, NHEJ repair pathway and early neural cell death, we used the embryonic mouse neuroretina, a well-established, classic model system of neural development^17^,^18^. Previous findings of our group have led to the description of an early phase of developmental cell death in the vertebrate retina that selectively affects recently born neurons^19^,^20^. In addition, we have performed an initial characterization of the occurrence of DSBs and of the requirement of active DSB repair during normal retinal development^21^. To support further the contribution of DSB repair to neural development, particularly that of the NHEJ pathway, we analyzed the Polμ^−/−^ mouse. The error-prone Polμ is a key component of the NHEJ repair system^22^,^23^,^24^. Polμ^−/−^ mice exhibit impaired somatic recombination of the immunoglobulin genes and defective hematopoietic homeostasis^25^,^26^. Moreover, Polμ-deficient mice show striking resistance to aging, which is selectively associated with significantly enhanced learning abilities and hepatic regenerative capacity in aged animals^27^,^28^. However, its impact on nervous system development has not been studied yet.

In the present work, we analyzed diverse retinal developmental processes in the Polμ^−/−^ mouse at E13.5, an early stage of neurogenesis. We found that Polμ deficiency affects neuronal viability and axonal growth, supporting that DSB repair is required for proper retinal development.

## Results

### Polμ is involved in DSB repair in the E13.5 mouse retina

**Figure 1:**
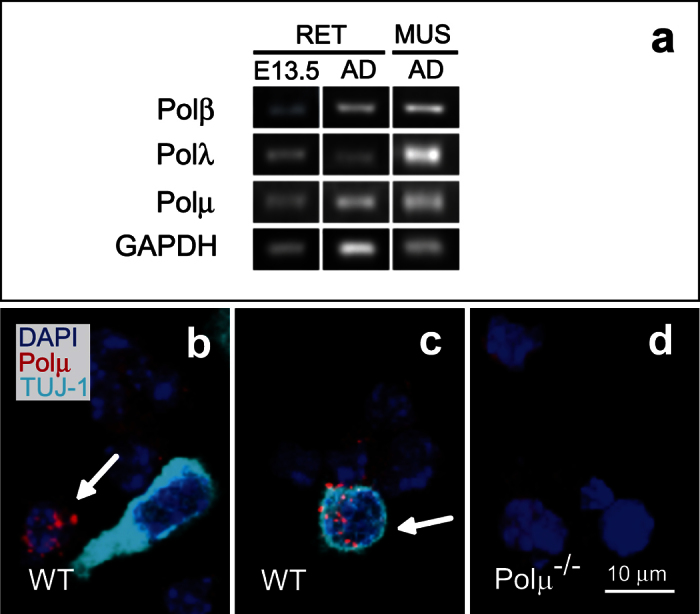
Polμ is present in E13.5 mouse retinas. **(a)** RT-PCR was used to detect mRNAs corresponding to the DNA repair polymerases β, λ, and μ in WT E13.5 and adult retinas and in adult muscle (AD, adult; RET, retina; MUS, muscle). **(b,c)** Polμ protein was detected by immunofluorescence in dissociated WT retinal cells. In some cases, Polμ nuclear foci (red; arrow) were present in cells displaying TUJ-1 neuronal staining (cyan). Cell nuclei were stained with DAPI (blue). **(d)** Dissociated Polμ^−/−^ retinal cells were used as a negative control.

Polμ expression and function were assessed to further examine the link between DSB repair and neurogenesis. Expression of Polμ and two other DNA repair polymerases, Polβ and Polλ, was initially determined by RT-PCR. mRNAs encoding these three enzymes were present in E13.5 retinas, when early neurogenesis is actively taking place, as well as in adult retina and muscle (Fig. 1a).

In addition to Polμ mRNA expression, dissociated E13.5 retinal cells were immunostained for Polμ (Fig. 1b,c). Polμ nuclear foci were present in 2.3 ± 0.29% of cells in WT E13.5 retinas (n = 9), mostly young neurons identified by immunostaining for the cytoskeletal protein βIII-tubulin (TUJ-1; Fig. 1b,c). The observed focal pattern is indicative of an active DSB repair process, as it has been described in lymphatic ganglia and in a lymphoma cell line^29^. By contrast, these foci were absent in Polμ-deficient cells (Fig. 1d).

**Figure 2:**
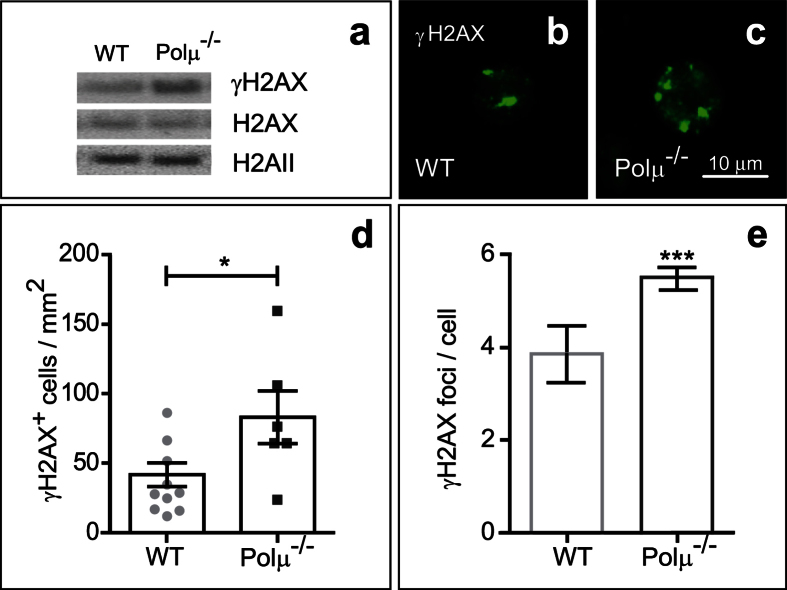
γH2AX expression is increased in E13.5 Polμ^−/−^ retinas. **(a)** Active DNA repair was evaluated by Western blot in WT and Polμ^−/−^ retinal protein extracts. γH2AX levels were compared with those of H2AX and H2AII. **(b,c)** γH2AX immunostaining (green) showed a focal distribution in WT and Polμ^−/−^ retinas. **(d)** The density of γH2AX-positive cells was quantified in whole-mount retinas. **(e**) The number of γH2AX-positive foci per positive cell was also scored in dissociated retinal cells (n > 100). Histogram shows the mean ± standard error of mean (s.e.m.), as well as the individual values in **(d)**. *P < 0.05 and ***P < 0.001 vs. corresponding controls.

The connection between Polμ expression and DSB repair during retinal development was investigated by determining the levels of phosphorylated H2AX histone (γH2AX), an early marker of DSB repair^30^. Western blot revealed increased levels of γH2AX in Polμ-deficient vs. WT retinas (Fig. 2a). γH2AX immunostaining revealed a classical focal distribution (Fig. 2b,c) both in WT and Polμ-deficient retinas, providing further evidence of the physiological occurrence and repair of DSBs in the developing retina. As expected in a model of impaired DSB repair, the number of cells with γH2AX foci, as well as the number of foci per cell, was higher in the absence of Polμ than in the WT retinas (Fig. 2d,e).

Taken together, these observations support the involvement of Polμ in naturally occurring DSB repair in the E13.5 mouse retina.

### Polμ deficiency affects neuronal viability in the E13.5 mouse retina

**Figure 3:**
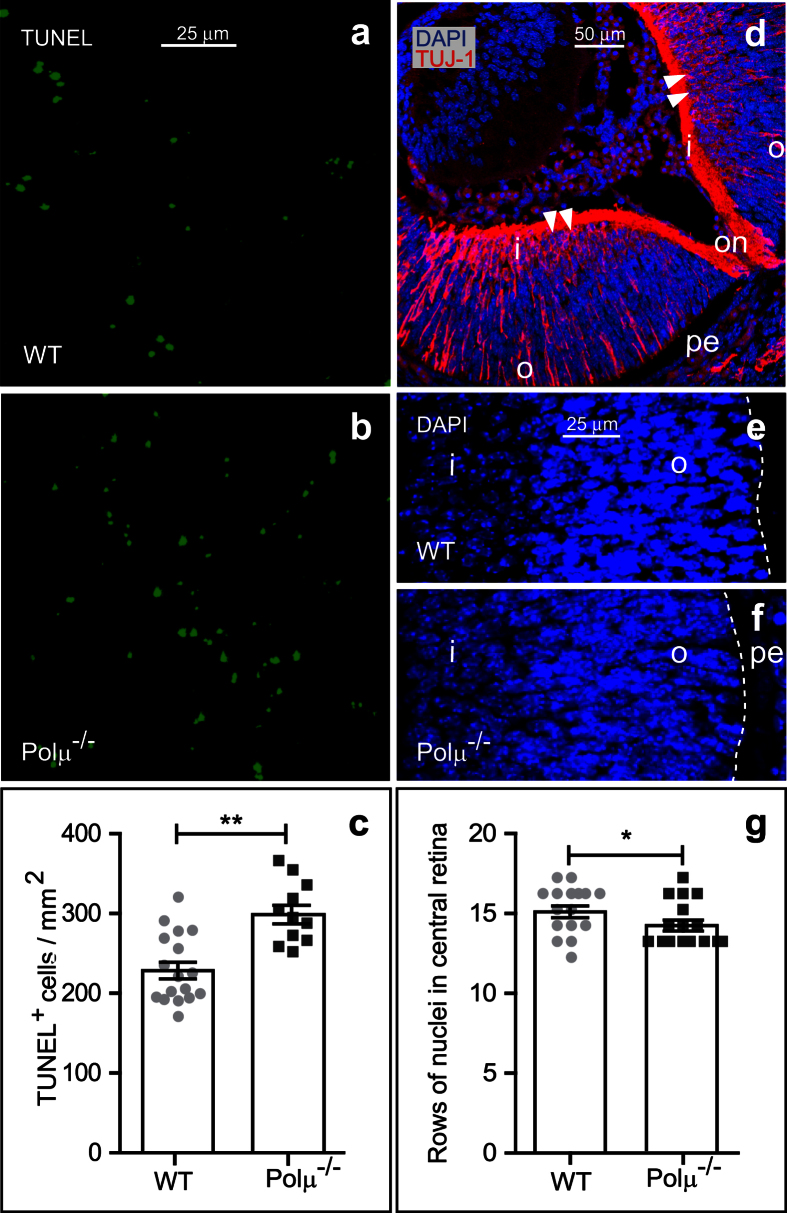
Programmed cell death is increased in E13.5 Polμ^−/−^ retinas. **(a,b)** Cell death in WT and Polμ^−/−^ whole-mount retinas was detected by TUNEL. **(c)** TUNEL-positive nuclei were quantified in whole-mount retinas. **(d–g)** Nucleus rows were scored in central retina sections stained with DAPI from WT and Polμ^−/−^ E13.5 mice. o, outer retina; i, inner retina; on, optic nerve; pe, pigmented epithelium, also indicated by a white dashed line. **(d)** An illustrative section, at low magnification, where the position of the scored rows are indicated by arrow heads. **(e,f)** Representative sections from WT and Polμ^−/−^ E13.5 mice retina, showing the nuclei staining. **(g)** Histogram shows nucleus row values corresponding to individual mice, together with the mean ± s.e.m. *P < 0.05 and **P < 0.01 vs. corresponding controls.

We next studied the consequences of Polμ deficiency on retinal development. The absence of Polμ was associated with an increase in the number of dying cells, as determined by scoring TUNEL-positive nuclei in E13.5 retinas (Fig. 3a–c). This increase in apoptosis also resulted in a modest but significant reduction in the number of nucleus rows in the central area of E13.5 retina sections (Fig. 3d–g).

**Figure 4:**
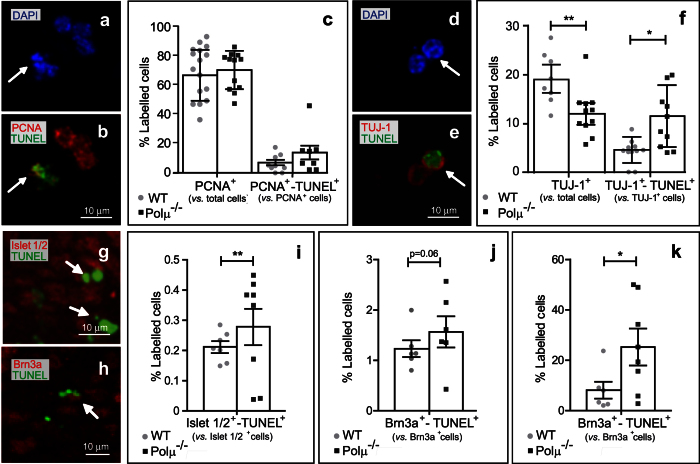
Neuronal death is selectively increased in E13.5 Polμ^−/−^ retinas. **(a–f)** Dissociated retinal cells from WT and Polμ^−/−^ animals were immunostained for PCNA (red, in **b**) or TUJ-1 (red, in **e**), and processed for TUNEL (green, in **b**,**e**) Nuclei were counterstained with DAPI (blue, in **a**,**d**). Numbers of total proliferative cells **(c)** and neurons **(f**), as well as those of doubly labelled cells, were scored. **(g,h)** Whole-mount retinas were immunostained for Islet1/2 (red, in **g**) or Brn3a (red, in **h**) and processed for TUNEL (green, in **g**,**h**), and labelled cells were scored. Arrow heads indicate doubly labelled cells. Simply and doubly labelled cells were scored (**i**,**j**). Dissociated cells were also immunostained for Brn3a and processed for TUNEL, and scored (**k**). Histograms show values corresponding to individual mice, together with the mean ± s.e.m. *P < 0.05 and **P < 0.01 vs. corresponding controls.

To identify the cells primarily affected by Polμ deficiency, different retinal cell subpopulations were analyzed. At E13.5 the mouse retina mostly consists of proliferating neuroepithelial cells, with a small proportion of recently differentiated neurons, including RGCs^17^. Dissociated retinal cells were doubly stained for TUNEL and either PCNA, a marker of proliferating neuroepithelial cells, or TUJ-1, a neuronal marker. While PCNA-positive cells were not significantly affected by Polμ deficiency (Fig. 4a–c), a decrease in the recently born TUJ-1-positive neuronal population was observed in the mutant retina (Fig. 4d–f). We confirmed the increase in young neuron cell death in the Polμ^−/−^ mouse by staining whole-mount retinas for the neuronal marker Islet 1/2, and TUNEL (Fig. 4g,i). A more precise identification of the nature of the dying neurons was achieved by Brn3a immunostaining, selective for RGCs (Fig. 4h,j,k). Whereas the limited staining of Brn3a dying neurons observed in whole-mount retinas only displayed a tendency to increase in the Polμ^−/−^ mouse, the larger amount of Brn3a dying cells detected in dissociated retinas, similar to that obtained with TUJ-1 (Fig. 4f), clearly demonstrated the selective death of young RGCs (Fig. 4k).

Our results indicate that Polμ deficiency selectively affects the survival of recently born neurons, including RGCs.

### Axonal navigation is disturbed in the Polμ-deficient mouse retina

**Figure 5:**
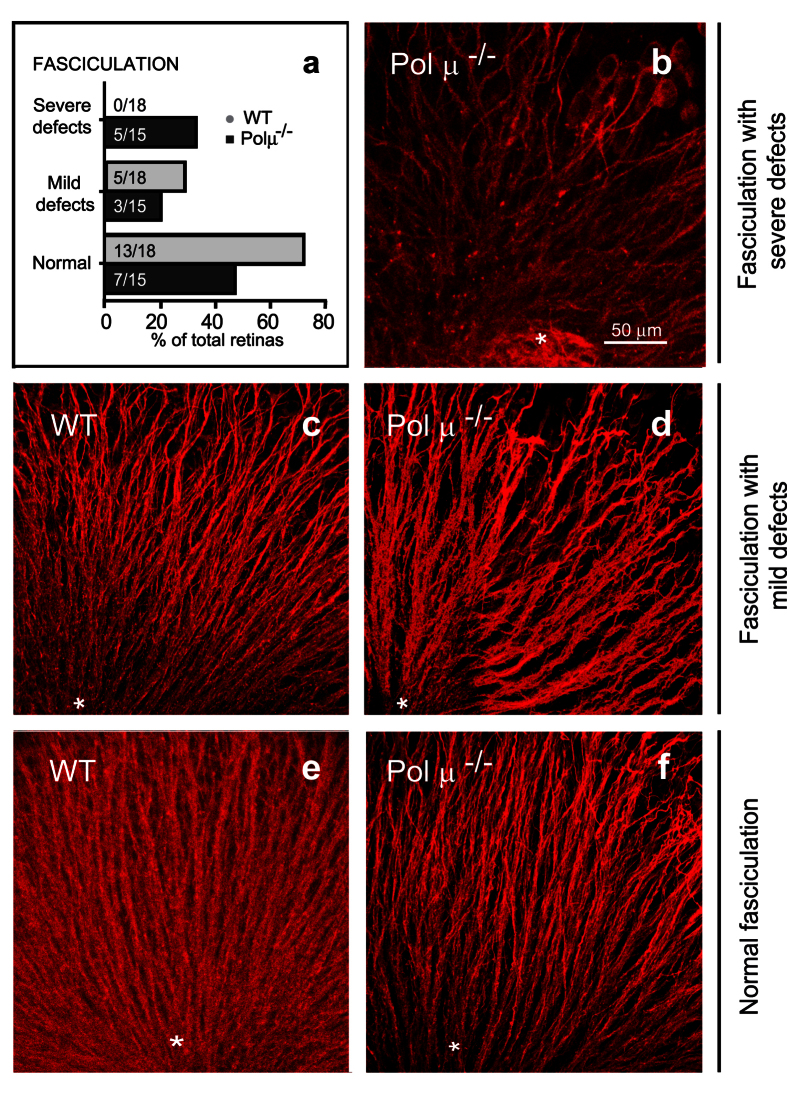
Intraretinal axonal trajectories are disturbed in E13.5 Polμ^−/−^ retinas. Whole-mount retinas were stained with TUJ-1 to visualize RGC axonal trajectories. Histogram **(a)** shows the proportion and the absolute number of retinas with each phenotype. **(b–f)** Representative dorsal retina fields in WT **(c**,**e)** and Polμ^−/−^ **(b,d**,**f)** mice. The asterisk marks the position of the optic nerve head. Axonal fasciculation was classified as severely defective **(b)**, mildly defective **(c**,**d)**, or normal **(e**,**f)**, and plotted accordingly **(a)**.

To further analyze the consequences of the lack of Polμ in the retina, we studied the course of retinal development, particularly that of RGCs. Shortly after differentiation, RGCs emit an axon that navigates towards the optic nerve head and the synaptic targets outside the retina. We visualized intraretinal axonal trajectories by TUJ-1 immunostaining in E13.5 whole-mount retinas and categorized them according to their regularity, ranging from normal fasciculation pattern to severe defects (Fig. 5a). In a significant number of cases, Polμ^−/−^ retinas showed profound disturbances in axonal growth, including marked defects in fasciculation, reduced axonal density, and aberrant, tangential trajectories (Fig. 5b). Conversely, WT retinas displayed a largely regular fasciculation array, centred on the optic nerve head (Fig. 5c,e) whereas Polμ-deficient mouse retinas frequently showed disordered trajectories, which were already evident in the least affected (i.e., normal fasciculation) group (Fig. 5d,f).

**Figure 6:**
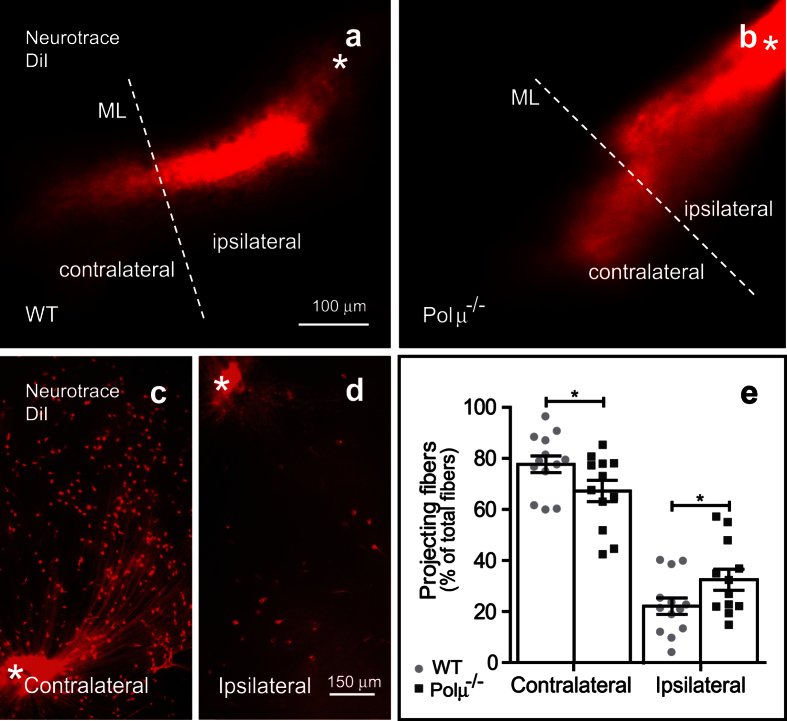
Midline cross-over and axonal decussation in Polμ^−/−^ retinas. **(a,b)** Midline cross-over in WT and Polμ^−/−^ animals was visualized at E13.5 using anterograde staining with DiI, from the retina towards the diencephalon midline (ML). The asterisk marks the position of the optic nerve head. Representative image of one of the 8 embryos analysed for each genotype. **(c,d)** Optic fibre decussation in WT and Polμ^−/−^ animals was visualized at E18.5 using retrograde staining with DiI, from the lateral geniculate nucleus towards the retina. **(e)** The proportion of fibres that reached the primary visual fields was quantified. Histogram shows values corresponding to individual mice, together with the mean ± s.e.m. *P < 0.05 vs. corresponding controls.

In route to the optic centres of the brain, most axons cross the midline at the optic chiasm. In the case of rodents, a small proportion of axons projects ipsilaterally. We visualized the path of RGC axons outside the retina by anterograde DiI labelling from the retina to the diencephalon to observe the forming chiasma. At E13.5, both WT and mutant mice retinal axons similarly crossed over the midline (Fig. 6a,b). A more detailed analysis of the primary visual centre innervation was performed by retrograde tracing at E18.5 from the lateral geniculate nucleus back to the RGC cell bodies (Fig. 6c,d). A modest but significant alteration in decussation, with an increased proportion of ipsilaterally projecting RGCs, was found in the Polμ^−/−^ mouse (Fig. 6e).

These results demonstrate that RGC axonal navigation is disturbed in the absence of Polμ.

### Disturbed axonal navigation in dissociated Polμ^−/−^ retinal cultures is cell autonomous

Proper axonal growth and navigation depend on the interplay between cell intrinsic mechanisms and environmental cues^31^. As an initial approach to characterize the relative contribution of cell intrinsic determinants to the Polμ^−/−^ axonal phenotype, isolated WT and Polμ^−/−^ retinal cells were cultured in absence of cell-to-cell contacts, in a homogeneous, defined environment, and were subsequently evaluated for their potential to emit normal neurites.

**Figure 7:**
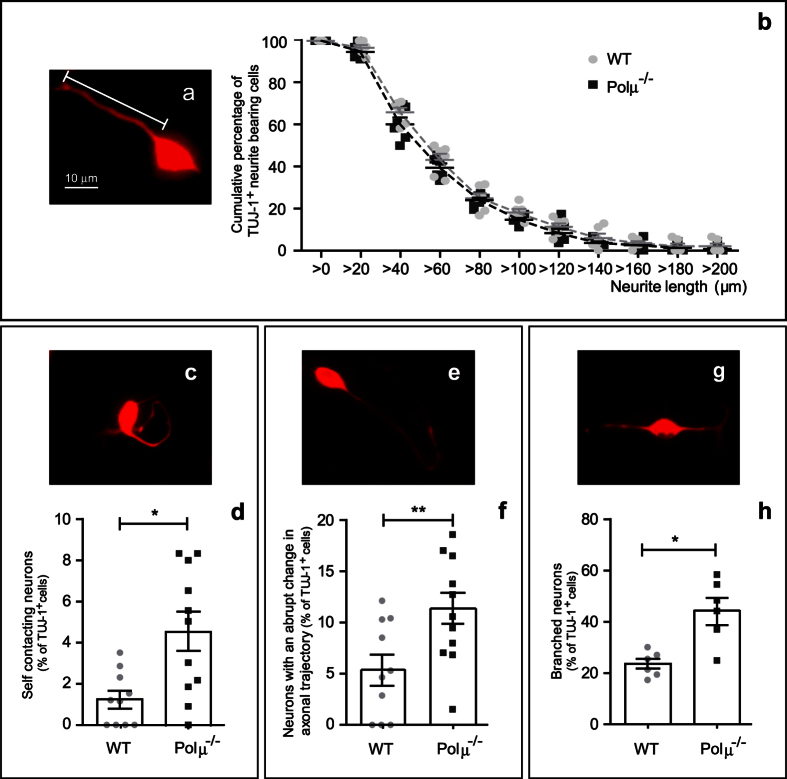
Neurite trajectories are disturbed in cultured E13.5 Polμ^−/−^ retinal cells. Dissociated retinal cells from WT and Polμ^−/−^ animals were cultured on laminin- and polyornithine-treated surfaces. Neurites were visualized by TUJ-1 immunostaining **(a,c,e**,**g)** and quantified by typology **(d,f**,**h)**. Neurite length was measured after 24 hours of culture **(b)**. Histograms show values corresponding to individual mice, together with the mean ± s.e.m. *P < 0.05 and **P < 0.01 vs. corresponding controls.

After 24 hr of culture, the proportion of neurite-bearing cells among isolated TUJ-1-positive cells (Fig. 7a) revealed no differences between WT and Polμ^−/−^ mice (84.7 ± 2.65% vs. 88.6 ± 3.0% of total cells, respectively; n = 6). Similarly, no differences were found in neurite length (Fig. 7b). Conversely, while most WT TUJ-1-positive cells emitted straight neurites (Fig. 7a; 66.7 ± 3.4% of labeled cells), this proportion decreased in Polμ^−/−^ cells (38.4 ± 7.5%). Consequently, morphologically aberrant neurites were more frequent in the mutant mouse (Fig. 7c–h). These anomalies included self-contacting neurites (Fig. 7c,d) and abrupt trajectory changes (Fig. 7e,f). Moreover, the number of TUJ-1-positive cells with branched neurites was increased in Polμ^−/−^ retinal neurons (Fig. 7g,h).

These observations suggest that the lack of Polμ causes a cell-autonomous defect in neurons, at least in culture, an observation in line with the aforementioned *in vivo* findings.

### Neuronal location and the distribution of L1-CAM and Bravo/Nr-CAM are altered in the E13.5 Polμ^−/−^ mouse retina

In addition to its impact on neuronal death, Polμ deficiency clearly affects axonal growth and navigation. Cell adhesion molecules of the immunoglobulin super-family (IgCAMs) are involved both in axonal guidance and neuronal migration^32^. Therefore, we analyzed the distribution of L1-CAM and Bravo, two members of the IgCAM super-family involved in axonal guidance during retinal development^33^, in order to identify a possible mechanism underlying the observed axonal disturbances.

**Figure 8:**
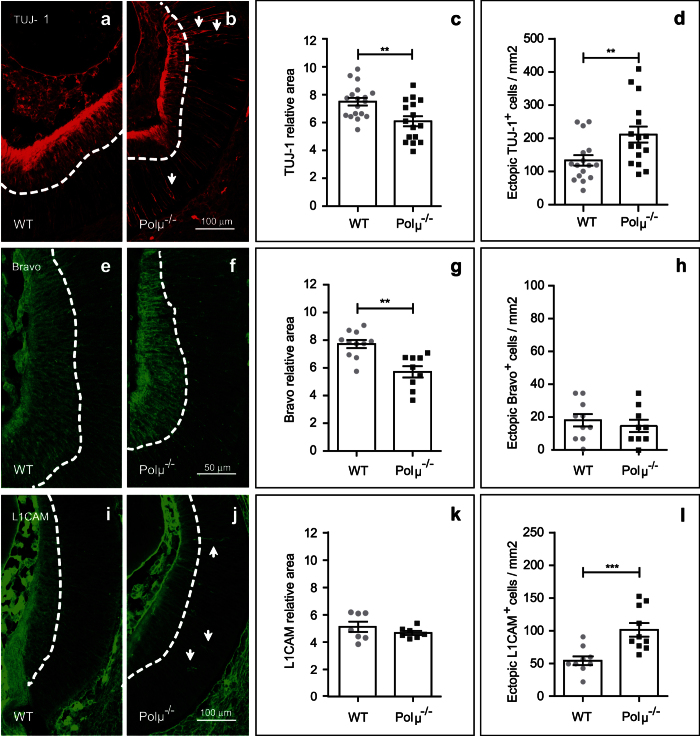
Distribution of cell adhesion molecules and neuronal location are altered in the E13.5 Polμ^−/−^ retina. Retinal sections from WT and Polμ^−/−^ mice were immunostained for TUJ-1 **(a**,**b)**, Bravo **(e**,**f)**, and L1-CAM **(i**,**j)**. The area occupied by TUJ-1 **(c)**, Bravo **(g)** and L1-CAM **(k)** immunostained cells was determined as described in the Materials and Methods and Supplementary Fig. 1. The number of ectopic neurons positive for TUJ-1 **(d)**, Bravo **(h)** and L1-CAM **(l)** was quantified. Histograms show values corresponding to individual mice, together with the mean ± s.e.m. **P < 0.01 and ***P < 0.001 vs. corresponding controls.

First, the distribution of TUJ-1 immunostaining was evaluated in E13.5 retinal sections from mutant and WT mice (Fig. 8a–d). This staining, which was selectively observed in both the soma and the axon of RGCs, delimited an area adjacent to the vitreal surface of the retina containing most of the RGC bodies that have already reached their definitive position. This area of staining was reduced in Polμ^−/−^ vs. WT retinas (Fig. 8c). Conversely, a higher number of ectopic TUJ-1-positive cells, outside the continuously stained area (see arrows in Fig. 8b) was observed in the mutant retina (Fig. 8d).

E13.5 WT and Polμ^−/−^ retinal sections were also immunostained for Bravo and L1-CAM and the resulting distribution patterns compared (Fig. 8e–l). Bravo staining was reduced in the mutant retina (Fig. 8g), correlating with TUJ-1 staining. By contrast, no differences in L1-CAM distribution were observed between WT and Polμ^−/−^ retinas (Fig. 8k). However, while Bravo staining revealed no differences in the number of stained ectopic cells between groups (Fig. 8h), a higher number of ectopic L1-CAM positive cells were observed in the mutant retina (Fig. 8l).

Taken together, our results reveal an additional effect of Polμ deficiency on RGC migration from the outer retinal surface, where terminal mitosis takes place, to the vitreal surface, their definitive position. This observation correlates with the altered distribution of the axonal surface proteins Bravo and L1-CAM. Our results support the genuine involvement of the polymerase Polμ, and consequently of the NHEJ DSB repair pathway, in neurogenesis and retinal development, coupled to the process of early neural cell death.

## Discussion

In this study we have analyzed the consequences of Polμ deficiency on different developmental processes in the retina. At E13.5, neurons, mainly RGCs, not only showed increased cell death in the Polμ^−/−^ mouse (Figs 3 and 4), but also exhibited defects in axonal growth and navigation (Figs 5, 6, 7) and impaired migration towards their definitive position in the retina (Fig. 8). These defects correlated with an altered distribution of the axonal guidance molecules L1-CAM and Bravo (Fig. 8), both of which are involved in RGC axon navigation^34^,^35^. We cannot rule out the possibility that some of the effects observed later in RGC development are merely a consequence of the initial death of RGCs. For instance, in zebra fish embryos, pioneer axons from early-born RGCs guide later-born RGC axons^36^. Some of the axonal growth disturbances described here may be caused by a lack of putative pioneer RGCs. However, the selective occurrence of DSBs during early RGC neurogenesis, which was also observed in WT animals (Fig. 2), and the cell-autonomous defects observed in isolated neurons (Fig. 7) require additional explanations.

The study of Polμ has significant advantages in comparison to previously studied DSB repair pathway components. Whereas DNA-PK^21^ may be involved in several processes, Polμ appears to be specialized in the NHEJ pathway. Furthermore, compared to the E13.5 lethal phenotype produced by disruption of the gene encoding DNA ligase IV^37^, a sealing enzyme required for the final step of NHEJ, Polμ deficiency gives rise to a mild phenotype that allows for a more detailed characterization. This mild phenotype may be in part due to the simultaneous availability of two NHEJ DNA polymerases: Polλ (Fig. 1a), which can rescue the joining of non-complementary ends if they are resected to present some eventual micro-homology^24^, and Polμ, specialized in rejoining DNA strands whose 3′ protrusions have minimal or even null complementarity, which can result in an error-prone outcome^24^,^38^,^39^. Interestingly, Polμ^−/−^ mouse mild phenotype is similar to those of mouse models carrying a hypomorphic ligIV allele with residual ligase activity, that presents increased apoptosis during embryonic forebrain formation^40^, and to the mouse model with diminished Nhej1 function, that shows cell death and neuronal migration defects that end up in cortical disorganization^41^.

The processes primarily affected in the Polμ^−/−^ mice are programmed V(D)J recombination of antigen receptor genes^25^,^42^, haematopoiesis^26^ and neurogenesis (the present study). This specificity indicates that in most Polμ^−/−^ non proliferative tissues, where homologous recombination is not available, Polλ activity may be sufficient to deal with DSBs. Although, according to our results, Polλ is present in the developing retina (Fig. 1a), the specific sensitiveness of neural and immune systems to Polμ deficiency suggests that they may accumulate more DSBs without terminal microhomology (non processable by Polλ) than other tissues, and opens the question of what process may cause such an imbalance in the type of DNA breaks generated in the nervous system.

The origin and nature of DSBs naturally occurring during neural development remains unclear. DNA damage may be a consequence of ongoing proliferation and high metabolic activity^15^. The selectivity of Polμ and the observations presented here in the Polμ deficient retina, however, suggest that the generation and repair of DSBs may display a genuine role in neural development as it does in the immune system. Indeed, this same correlation also appears in human patients carrying defects in NHEJ proteins that present severe immunodeficiency and additional developmental abnormalities, like microcephaly (see^43^ for review).

We and other colleagues have speculated that neurogenesis concurs with the generation of somatic mosaicism among projecting neurons, in a manner similar to the somatic recombination in the immune system^2^,^44^,^45^. In 1977, Hood and co workers^46^ proposed that cell recognition processes, such as that mediating the connectivity of RGC axons with their targets, may depend on the generation of varied guidance cues. This diversity may be obtained through different processes, including genetic diversification. Strikingly, the distribution of axonal guidance molecules appears to be altered in the Polμ^−/−^ mouse. One simple explanation is that the accumulation of unresolved DNA breaks in Polμ^−/−^ mutant mice may promote genomic reorganizations^8^,^13^ that could lead to apoptosis if not properly repaired. However, observing Polμ^−/−^ phenotype (Fig. 4 and^23^,^26^,^27^) we cannot exclude a specific role for Polμ within the neuronal subpopulation. Although up to now there are no proofs of site-directed mechanisms similar to somatic recombination in the nervous system, whole genome amplification techniques have revealed the existence of somatic mosaicism in the vertebrate brain, including the human brain^47^,^48^,^49^,^50^,^51^. Indeed, single-cell sequencing has shown that neurons accumulate mosaic copy number variations (CNVs) and show higher retrotransposition rates during neurogenesis^48^,^51^,^52^,^53^,^54^. Recent studies show that human neurons accumulate single-nucleotide somatic variants^55^, which, together with other factors like epigenomic modifications and transcriptional regulation, will contribute to a huge neuronal diversity^56^. It is thus tempting to propose that the activity of an error-prone DNA polymerase, such as Polμ, may indeed promote the accumulation of small changes in the neuronal genome, potentially contributing to neuronal genetic diversity.

In summary, the Polμ^−/−^ mouse retina has enabled the characterization of the impact of a mildly impaired NHEJ pathway in the proper generation and the connectivity of neurons, providing new clues to the possible functional role of DSB generation and repair. Furthermore, future research including single neuron sequencing may eventually determine the precise role of NHEJ pathway in neuronal diversification, as it has in the immune system.

## Materials and Methods

### Mice

C57BL/6J mice (WT) were obtained from Harland (Gannat, France). Polμ^−/−^ mice were generated on a mixed 129/Balb/c background^26^,^57^ and back-crossed (>10 times) to a C57BL/6 background. Polμ^−/−^ and WT mice were bred in local facilities. Male and female mouse embryos and adult tissues were obtained from euthanized animals of the indicated ages. All experimental procedures were approved by the CSIC bioethics committee for animal experimentation and the Dirección General de Medio Ambiente, Comunidad de Madrid, and performed in accordance with European Union regulations for the use and treatment of animals in research (RD53/2013, BOE, Spain).

### RT-PCR analysis

Freshly dissected tissues were frozen on dry ice. Total RNA was extracted using Trizol reagent and DNase I (Invitrogen, Carlsbad, CA, USA). Reverse transcription of 2.5–5 μg of total RNA was performed using Oligo(dT)_18–20_ primer and the Superscript III enzyme (Invitrogen). Semi-quantitative RT-PCR was performed on 1–2 μL of the reverse transcription product using Taq polymerase (Invitrogen), according to the following protocol: 94 °C for 1 min, 35–38 cycles of denaturation (26 cycles for GAPDH primers) at 94 °C for 30 s, 58 °C for 45 s; 72 °C for 45 s; and a step at 72 °C for 1 min. The following primer sequences were used: Polμ (Fw, TCAGAGGGCTCTGGAGACGCTA; Rv, CCTACTTCTTGCCCCTCCTC); Polλ (Fw, GCAGCCAGAAGGCAACTAAC; Rv, AGCTCCAAGACAGGCACACT); Polβ (Fw, CCATCTGCTGCAAGGAAGT; Rv, GCTGGATTCTGACGTGAAG); and GAPDH (Fw, GCAATGCATCCTGCACCACC; Rv, AGTGATGGCATGGACTGTGG). RT-PCR products were visualized as described^21^.

### Western blotting

Individual retinas were lysed, resolved and transferred to PVDF membranes and blocked as described^21^. Membranes were incubated with antibodies against γH2AX (1/1000, Abcam #ab22551#, Cambridge, UK); H2AX (1/5000, Abcam #ab11175#); H2AII (1/1000, Cell Signaling Technology #2578#, Danvers, MA, USA), and β-tubulin clone TUB 2.1 (1/10000, Sigma-Aldrich #T4026#, Steinheim, Germany). The membranes were developed as described^21^.

### Immunostaining of dissociated cells

Dissociated cells from freshly dissected retinas were prepared and processed as described^21^. Cytospin slides were incubated overnight at 4 °C with primary antibodies against human Polμ protein (1/200)^29^; β-III tubulin clone TUJ-1 (1/1000, Covance #PRB-435P#, Paris, France); γH2AX (1/1000, Ser 139, Abcam #ab22551#, Cambridge, UK); Brn3a (1/50, Millipore-Merck #AB1585#, Darmstadt, Germany) and PCNA (1/250, Delta Biolabs #DB051:PCNA(C19)#, Gilroy, CA, USA). Slides were incubated for 1 h at RT with Alexa Fluor series conjugated secondary antibodies (1/250–1/500, depending on the primary antibody; Molecular Probes, Thermo Fisher Scientific, Rockford, IL, USA), and mounted with Fluoromount-G mounting medium (Southern Biotech, Birmingham, AL, USA). Cell staining and scoring were performed using a fluorescence microscope (Zeiss Axioplan, Oberkochen, Germany) coupled to a CCD camera (Leica, Wetzlar, Germany) or a confocal microscope (Leica TCS-SP5-A0BS) with a 40X objective, analyzing 100–500 cells from non-adjacent fields.

### Immunostaining of retinal sections

Entire heads from E13.5 embryos were fixed with 4% (w/v) PFA in 0.1 M phosphate buffer, pH 7.4 for 24 h, and cryoprotected by subsequent immersion in 15% and 30% sucrose (w/v, Sigma-Aldrich) in 0.01 M phosphate buffer, pH 7.4 for 24 h each. Samples were embedded in OCT compound (Sakura Finitek Europe, Zoeterwoude, Netherlands) and frozen at −80 °C until use. Horizontal retinal sections (10 μm thick) were cut on a cryostat (Leica) and collected on Superfrost slides (Menzel-Glasser, Braunschweig, Germany). Cryostat sections were refixed for 20 min with 4% (w/v) PFA in 0.1 M phosphate buffer, pH 7.4. After permeabilization in 0.2% (w/v) Triton X-100, sections were blocked with 15% (v/v) normal goat serum (Sigma-Aldrich) and 0.2% (w/v) Triton X-100. Retina slices were incubated overnight at 4 °C with primary antibodies against β-III tubulin (1/1000), L1-CAM (1/500, Abcam #ab24345#, Cambridge, UK), and Bravo (rabbit polyclonal; 1/250)^33^. Samples were incubated for 1 h at RT with Alexa Fluor series conjugated secondary antibodies (1/250–1/500, depending on primary antibody), and mounted with Fluoromount-G mounting medium. Cell staining and cell distribution were analyzed by confocal microscopy; confocal images of 4–6 slices per retina were obtained using a 40X objective.

### Quantification of nucleus rows in central retina

Four different nucleus rows were counted in central retina slices counterstained with 4′,6-diamidino-2-phenylindole (DAPI; 1 μg/mL, Sigma-Aldrich; Fig. 3d). Counting was performed at 4–6 nuclei of distance from the optic nerve head in at least four slices per animal.

### Quantification of immunostaining and ectopic cells in retinal sections

Confocal images of retinal slices were acquired using constant intensity settings (Supplementary Fig. 1). The areas comprised of TUJ-1, Bravo, or L1-CAM-positive cells were automatically detected using the Isolines surface plot tool from FIJI software^58^. The following settings were used for TUJ-1-positive cells: original colours; grid size, 512; smoothing, 40.0; perspective, 0.0; lighting, 0.36; scale, 1.00; Z-scale, 0.77; max, 67%; min, 5%. For detection of Bravo and L1-CAM staining, the following parameters were used: mesh; original colours; grid size, 256; smoothing, 10.0; perspective, 0.16; lighting, 32; scale, 1.02; Z-scale, 0.81; max, 94%; min, 5%. The largest continuous area of immunostaining within the neuroretina was measured with FIJI software. Cells lying outside this region were considered ectopic and were scored individually.

### Immunostaining of whole-mount retinas

Freshly dissected neuroretinas were flat-mounted onto nitrocellulose membranes and fixed, permeated and blocked as previously described^21^. Retinas were incubated overnight at 4 °C with primary antibodies against γH2AX (1/1000), β-III tubulin (1/1000), Islet-1/2 (1/200, Developmental Studies Hybridoma Bank #39.4D5#, Iowa, IN, USA) and Brn3a (1/50). The retinas were incubated for 1 h at RT with Alexa Fluor series conjugated secondary antibodies, and mounted with Fluoromount-G mounting medium. Cell staining and cell density were analyzed by confocal microscopy, using 3–4 confocal images per retina (depending on retina size) obtained with a 40X objective. γH2AX staining was evaluated using fluorescence microscopy. Cell density was calculated based on the number of cells present in the confocal images and the size of the imaged area. All determinations were made using FIJI software.

### Detection of apoptosis

The TUNEL (TdT-mediated dUTP nick end-labelling of fragmented DNA) technique was performed as previously described^59^. Samples were counterstained with DAPI, mounted on a slide with Fluoromount-G mounting medium and analyzed under a fluorescence microscope. The density of TUNEL-positive cells was determined by counting the labelled nuclei throughout the entire retina using a 100X objective. Images from whole-mount retinas were obtained by confocal microscopy.

### Anterograde DiI staining

Eye lens and neuroretinas, preserving the pigmented epithelium, were dissected out from E13.5 embryo heads, which were subsequently fixed for 28 h at 4 °C with 4% (w/v) PFA in 0.1 M phosphate buffer, pH 7.4. Neurotrace DiI (Molecular Probes) was deposited over the optic nerve head and the heads were maintained for 2 weeks at 37 °C in PBS containing 0.1% (w/v) sodium azide. Axonal midline crossing was visualized by confocal microscopy in a vibratome section of the head containing the retinas, optic nerve and optic chiasm.

### Retrograde DiI staining

E18.5 embryo heads, after removal of skin, cranial bones and meninges, were fixed for 48 h at 4 °C with 4% (w/v) PFA in 0.1 M phosphate buffer, pH 7.4. The cerebellum, medulla oblongata and telencephalon were dissected. Neurotrace DiI was deposited within a deep incision in the ventral geniculate body or posterior optic tract. Heads were incubated for 3 weeks at 37 °C in PBS containing 0.1% (w/v) sodium azide. Neuroretinas were subsequently dissected from the surrounding tissues. DiI-positive ganglion cell bodies were visualized in whole-mount retinas on a fluorescence microscope.

### Primary dissociated neuroretina cell culture

Neuroretinas were collected from E13.5 embryos, pooled from the whole litter and dissociated for 10 min at 37 °C in 0.05% (w/v) trypsin (Worthington Biochemical Corporation, Lakewood, NJ, USA) in PBS containing 3 mg/mL BSA. Disaggregation was stopped by adding 0.1% (w/v) soybean trypsin inhibitor and 25 μM DNase I (both from Sigma-Aldrich). Cells were counted and incubated in chemically defined DMEM/F12 medium (Gibco, Life Technologies, Rockford, IL, USA) with N2 supplement, as previously described^60^. Cells were plated at an estimated density of 85,000 cells/cm^2^ in 2-cm^2^ Permanox chamber slides (NUNC, Thermo Fisher Scientific) previously treated for 1 h with 0.5 mg/mL polyornithine (Sigma-Aldrich) in 0.15 M sodium borate [pH 8.35], and for 1 h with a PBS solution containing 1 μg/mL laminin (Sigma-Aldrich), and 0.1% (w/v) BSA. Cells were incubated for 18–24 h at 37 °C and 5% CO_2_. After culture, the cells were fixed for 20 min with 4% PFA in 0.1 M PB, pH 7.4 at 4 °C, and processed for immunostaining as described above for dissociated cells.

## Statistical analysis

Data size was estimated in accordance with previous literature. All experiments were run at least twice, with several animals from at least 3 independent litters. Randomization method was not applied because experimental groups consist in different genotype litters. No blinding was performed in any experimental procedure. Data points in graphs represent individual mice, and bars in all panels represent the mean and the standard error of the mean (s.e.m.).

Data were checked for normality using both D’Agostino-Pearson omnibus and Shapiro-Wilk normality tests sequentially. Data were considered to fit a normal distribution only if they passed simultaneously both tests. For normal data, Fisher’s test was used to determine whether the variance of the samples analyzed was comparable (homoscedasticity). Normal data were compared using an unpaired Student’s T-test, applying Welch’s correction in cases of non-homoscedasticity. In cases of non-normal samples, populations were compared using the Mann Whitney nonparametric U-test. Outliers were detected by Grubbs’ outlier test, and excluded from further analysis. If previous literature allowed us to make a prediction about the result of the experiment, then one-sided test were applied. Otherwise, tests were two-sided. All analyses were performed at a fixed 95% confidence interval, using GraphPad Prism version 5.01 for Windows (GraphPad Software, San Diego, CA, USA; www.graphpad.com). Statistically significant differences are indicated as follows: *p < 0.05; **p < 0.01; ***p < 0.001.

## Additional Information

**How to cite this article**: Baleriola, J. *et al.* Increased neuronal death and disturbed axonal growth in the Polµ-deficient mouse embryonic retina. *Sci. Rep.* **6**, 25928; doi: 10.1038/srep25928 (2016).

## Supplementary Material

Supplementary Figure 1
