# Supplementary material for: Increased neuronal death and disturbed axonal growth in the Polμ-deficient mouse embryonic retina
Source: Sci Rep. 2016 May 13;6:25928. doi: 10.1038/srep25928 (PMC4865816; doi:10.1038/srep25928)

SUPPLEMENTARY INFORMATION

Increased neuronal death and disturbed axonal growth in the Polμ-deficient mouse embryonic retina.

Jimena Baleriola, Noemí Álvarez-Lindo, Pedro de la Villa, Antonio Bernad, Luis Blanco, Teresa Suárez and Enrique J. de la Rosa

Supplementary Figure S1. Area detection.

The retinal area occupied by TUJ-1, Bravo or L1-CAM stained cells was automatically detected using the Isolines option from the 3D surface plot tool in FIJI software. Original confocal images **(a)** were sequentially transformed **(b, c and d)**. Areas were determined in the top view projection **(d)**. Cells lying outside the determined area were considered ectopic.

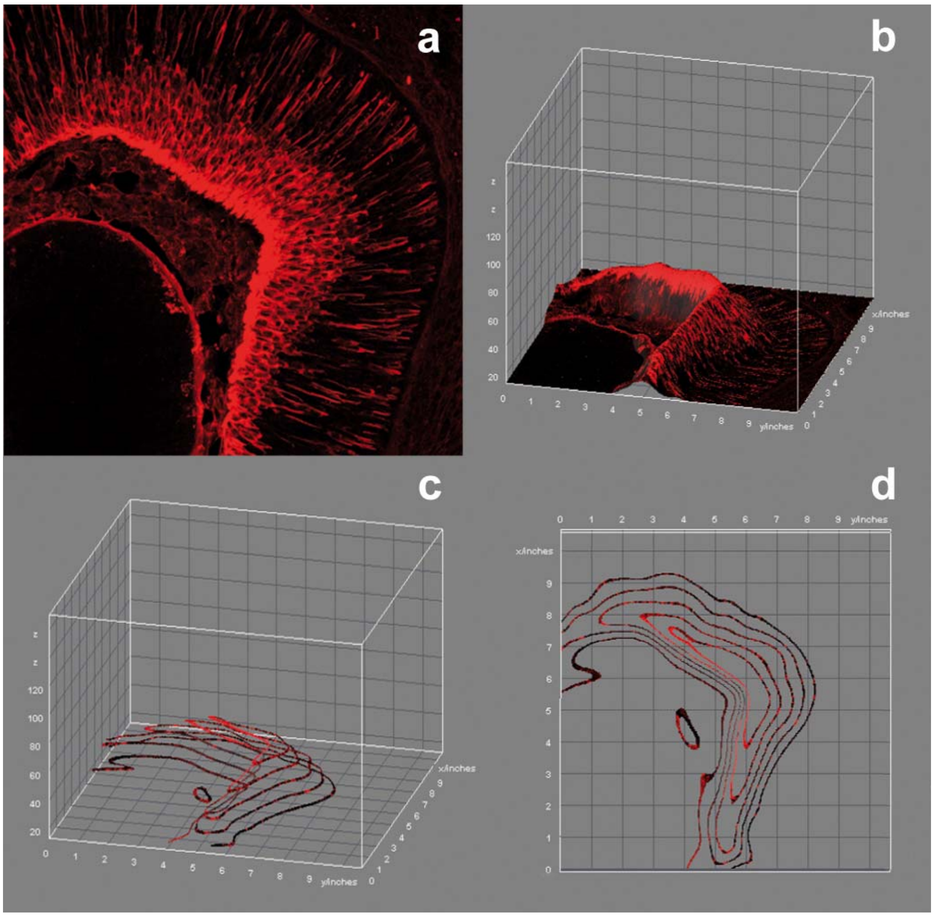

Supplement: Supplementary Figure 1 [file srep25928-s1.pdf]
